# A Mixture of Full-Fat and Defatted *Hermetia illucens* Larvae and Poultry By-Products as Sustainable Protein Sources Improved Fillet Quality Traits in Farmed Barramundi, *Lates calcarifer*

**DOI:** 10.3390/foods12020362

**Published:** 2023-01-12

**Authors:** Md Reaz Chaklader, Wing H. Chung, Janet Howieson, Ravi Fotedar

**Affiliations:** 1 School of Molecular and Life Sciences, Curtin University, 1 Turner Avenue, Bentley, WA 6102, Australia; 2 Department of Primary Industries and Regional Development, Fleet Street, Fremantle, WA 6160, Australia

**Keywords:** black soldier fly larvae, sensory evaluation, muscle microstructure, texture and color, lipid oxidation

## Abstract

The physicochemical quality and shelf-life of fillets from barramundi, which were fed for 56 days on a mixture of poultry by-product meal (PBM), full-fat *Hermetia illucens* (FHI), and defatted HI (DHI), were investigated and compared to a fishmeal (FM) control diet. The proximate and total amino acids compositions of the fillets were unaffected by the test diets, while the mixture of PBM and HI larvae improved the sensory quality. An eight-day shelf-life study showed that PBM-HI-based diets improved the texture profile based upon the chewiness, cohesiveness, gumminess, and hardness, regardless of the storage time. The improved texture was aligned with comparatively less degradation of the microstructure of the muscle tissue in the same diets. An improvement in the quality index (QI) value, an increase in pH, and a decrease in lipid oxidation were also found in the fillets of barramundi fed test diets compared with the control diet during the storage time. The test diets positively influenced flesh lightness and redness, while the color profiles were negatively influenced by the storage time. Overall, the maintenance of compositional attributes; the enhancement of fillet sensory attributes, texture, and brightness; and the improved raw fillet shelf-life support the inclusion of PBM-HI-based diets in aquafeed.

## 1. Introduction

In human nutrition, fish are an important source of lipids, particularly omega-3 polyunsaturated fatty acids (PUFA), providing vitamins and trace elements. The regular consumption of fish at least once a week is highly recommended, as it improves neural development in infants and prevents chronic illnesses such as hypertension and cardiovascular disease [1,2,3,4,5]. The plateau in the volume of captured landing fisheries has resulted in an expansion of aquaculture to meet these nutritional benefits in humans. Aquaculture now provides 52 percent of fish consumed by humans [6].

Barramundi, *Lates calcarifer*, also commonly known as Asian seabass, is an important carnivorous fish species in aquaculture due to its rapid growth, high nutritional value, and adaptation to freshwater and seawater environments. The barramundi production reached 76,843 tonnes with a global value of USD 320 million in 2018 [7,8]. Barramundi require a high protein intake, which is largely obtained from fishmeal (FM). However, while aquaculture is projected to increase, FM in carnivore aquafeed production peaked in 1980 at an estimated 6.3 million metric tons annually [9,10]. Consequently, barramundi farming has increasingly relied on FM, which is reducing its profitability [11].

Aquaculture nutrition studies on barramundi diets have been conducted since at least 1980 to investigate suitable alternative protein ingredients to replace FM [12]. Very few studies have described and highlighted the importance of linking aquaculture nutrition studies using alternative, sustainable ingredients and focusing on fish growth and physiological response, with a food science investigation of the aligned edible fillet. For example, poultry by-product meal (PBM), an important alternative protein ingredient, has a similar protein and amino acid profile to FM. PBM is readily available at a relatively low cost and might become a replacement protein source for carnivorous aquafeed production [13,14,15,16]. PBM has been investigated in the diets of a wide variety of finfish and shellfish, with variable outcomes [17]. Several recent studies have reported total FM substitution by PBM together with fish protein hydrolysate and insect larvae [18,19,20,21], with positive outcomes on growth, physiological, and immunological responses; however, food science attributes of the fillet products were not described.

The 2017 approval of insect meal in aquadiets by the European Union (Commission Regulation (EU) 2017/893) [22] has stimulated research on replacing FM with insect meal. Insect meal has several environmental and economic benefits primarily because of the fast growth and reproduction rates, minimal requirement for water and land, and capability to biodegrade low-quality waste into high-value protein ingredients. The frass (excrement) can also be used as a soil ameliorant [23,24,25].

The larval stage of *Hermetia illucens* (HI), commonly known as black soldier fly, has been regarded as promising for inclusion in aquafeeds, as it contains comparable protein (~60%) and amino acids to FM. It is also a good source of energy (10–30% lipids), vitamins, and minerals [26], and contains functional molecules, including antimicrobial peptides and novel polysaccharides, such as silkose and dipterose [18,21]. The nutrient compositions in HI larvae may vary depending on the process, stage of growth, and substrate, and may also change during processing, such as in defatting. While defatting may allow a higher inclusion of HI meal in the diet, the process increases the chitin ratio, causing a negative effect on the growth of finfish such as barramundi [15]. The inclusion of HI larvae meal (both full-fat and defatted) in the diet of several fish species have been evaluated at various inclusion levels without negative effects on growth and other physiological responses [27,28].

Both HI and PBM as alternative protein ingredients in developing sustainable aquafeeds have been quite commonly investigated. However, while the growth and physiological aspects of the fish are well documented, many of these studies have largely overlooked the food science aspect of the aligned fillets. Such food-science-based investigations are important when introducing a new protein source into fish feed [29], since the fillets’ sensory characteristics as well as physical and chemical compositions could be influenced by the dietary ingredients [30,31,32]. Furthermore, for industrial applications, it is important to know the potential effect of a new aquafeed ingredient, particularly that the fillet quality and consumer or market acceptance will not be compromised.

Such food science investigations comparing the impacts of alternative aquafeed ingredients can include relevant compositional and nutritional aspects such as the amino acid and fatty acid profiles, physical aspects such as the fillet color and texture, as well as the organoleptic or sensory aspects of the edible product [33].

A recent study reported that feeding with full-fat HI larvae meal (6.5 and 19.5%) did not affect the fillet color in juveniles of European seabass, *Dicentrarchus labrax* [20]. However, the inclusion of 25 and 50% of HI meal significantly affected the sensory profile of fillets of rainbow trout, *Oncorhynchus mykiss* [30]. Llagostera et al. [34] evaluated consumer perceptions towards fish fed on insect meal and reported that consumers could not discern the fish fed with insect meal. These discrepancies highlight the importance of a hedonic evaluation (sensory test) of edible quality parameters to fully understand consumers’ preferences, perceptions, and acceptance of new alternative protein ingredients in aquafeed.

The fillet quality of farmed fish may be influenced by the feed ingredients and post-harvest processing and storage methods [35,36,37,38]. The post-harvest storage time is associated with oxidative, enzymatic, and bacterial processes that can degrade the flavor, taste, and aroma qualities and promote changes to the texture, color, pH, quality index, and nutritional status. Negative outcomes lead to final fish products that are unfit for marketing and consumption [37,39,40,41,42]. Hence, the investigation of new aquafeed ingredients could also include some shelf-life assessments of a range of relevant chilled retail display parameters, resulting in the definition of retail display and end-user storage recommendations. The tested shelf-life fillet parameters include a range of physical (texture, color, drip loss, and quality index), biochemical (pH and lipid oxidation analyses), and microbiological attributes and their interactions [43].

In the particular case of HI inclusion in aquafeeds, enriching tissues with functional materials, which prevented lipid oxidation in barramundi muscles during chilled storage, has been reported [37]. In this context, the antioxidant properties of HI larvae are well documented in terms of reducing the lipid oxidation in the muscles of juveniles of European seabass [22] and rainbow trout [32] during storage.

Hence, the present study was a first attempt to understand if the complete protein source inclusion of fatted or defatted HI larvae (particularly when produced partially on fish processing outputs) in combination with PBM in barramundi diets would positively or negatively influence the compositional, physical, and sensory quality traits and shelf life of fillets when compared to products from traditional FM-based diets.

## 2. Materials and Methods

### 2.1. Ethical Statement, Diets, Animal Husbandry, and Fish Sampling

The experimental procedures for fish handling and care during the fish trials with the alternate diets were in strict compliance with the guidelines and regulations of Australia. The experimental protocols were reviewed and approved by the Curtin University Animal Ethics Committee (ARE2018-37).

The experimental design, diet formulation, and animal husbandry protocols were reported in our previous publication [15]. Briefly, three test diets containing identical proximate composition were formulated to test an FM-based diet as the control (0PBM-0HI) and two test diets with complete FM replacement by 70 + 30% of PBM and FHI (70PBM-30FHI) and partially DHI (70PBM-30DHI). The rearing process of *Hermetia illucens* (black soldier fly) on a mixture of carp mince and grain waste (70:30) for 6 days was as previously described [21]. The ingredient composition and amino acid composition profiles of the test diets and ingredients such as the PBM, FHI, and DHI are presented in Table 1.

To perform the trial, 225 fish with similar weights were stocked into nine tanks with 25 fish per tank and fed diets in triplicate. After a 56-day feeding trial, the fish were starved for 24 h and eight fish per tank were sacrificed by euthanasia (AQUI-S, 175 mg/L), six fish per tank (18 fish/treatment) were used for the fillet quality traits analysis, and two fish per tank (6 fish per treatment) were used for the amino acid analysis.

Among the samples allocated for fillet quality analyses, all fishes were filleted, by a trained researcher to ensure consistency between samples, who left the skin on the fillets. For each fish, one of the fillets was subjected to individual quick freezing (IQF) via immersion in liquid nitrogen with constant stirring, then vacuum-packed and stored immediately at −80 °C until the commencement of sensory evaluation. Another fillet was packed in a labelled resealable polyethylene bag on ice and transported to the simulated retail display storage area within one hour.

### 2.2. Proximate Composition

#### 2.2.1. Crude Protein

Crude protein analyses were conducted as per the AOAC [44] method. In brief, 1 g of the sample was weighed and placed along with a Kjeldhal catalyst tablet and glass bead in a digestion tube. Next, 8 mL of sulfuric acid and phosphoric acid was added, followed by the addition of 4 mL of 35% hydrogen peroxide mixture to each tube. All tubes were placed in a Kjeltec digester block (Foss Tecator 2020, Hoganas, Sweden) for digestion at 420 °C. To determine the nitrogen content, the digested sample was distilled using a Kjeltec distilling unit (Foss Tecator 1002, Högänas, Sweden). The distillate was collected into a flask with 25 mL of boric acid containing bromocresol green and methyl red indicators. Next, titration with hydrochloric acid was conducted on the final distillate, and a conversion factor of 6.25 was used for the calculation of the crude protein content.

#### 2.2.2. Crude Fat

The petroleum ether extraction method described in the AOAC [44] was used to determine the crude fat content. A Soxhlet unit (extraction unit E-816, BÜCHI Labortechnik AG, Flawil, Switzerland) was used to extract fat from the samples. The extracted fat was then dried at 105 °C until a constant weight was achieved, and the crude fat percentage was calculated by dividing the weight of the extracted fat by the weight of the raw material fat.

#### 2.2.3. Ash

The AOAC [44] ash determination method was used in this study. The sample was weighed prior to and after heating at 550 °C overnight using a muffle furnace (Thermolyne muffle furnace, model 48000, Thermo Fisher Scientific Inc., Waltham, MA, USA). The ash content was calculated by dividing the post-ashing sample weight by the initial sample weight.

#### 2.2.4. Moisture

The AOAC [44] standard method was used to determine the moisture content of the samples. The sample was weighed before and after oven drying until reaching a constant weight at 105 °C. The moisture content was then calculated by dividing the post-drying weight by the pre-drying weight.

### 2.3. Amino Acid Analysis

An amino acid profile of the test diets, DHI larvae, and barramundi muscle samples was performed following the standard protocol of the Australian Proteome Analysis Facility (APAF), Macquarie University, Sydney NSW 2109. The amino acid analysis was performed using liquid hydrolysis in 6M HCl to release the amino acids from the protein, followed by quantification using the pre-column derivatization reversed phase (RP) HPLC procedure. The samples were weighed out in duplicate or singularly into hydrolysis vials, and 5 mL of 20% HCl was added. These were then incubated at 110 °C for 24 h. After hydrolysis, the samples were derivatized using the AccQTag reagent (Waters Corporation, Milford, MA, USA) [45,46] and then analyzed using a high-resolution RP column on a UPLC system with 10 min run times. The instrument used was an ACQUITY UPLC system with a UV detector (Waters Corporation, Milford, MA, USA) [47]. For all analyses, a Waters AccQTag Ultra column (BEH C18, 2.1 mm × 100 mm; 1.7 µm) was used [48]. The column temperature employed was 57 °C, the detection was performed at 260 nm, and the flow rate was 0.7 mL/min. This method does not analyze tryptophan or cysteine, which are acid-sensitive and require special conditions for their analysis.

### 2.4. Sensory Quality

The sensory evaluation was designed based on Gedarawatte et al. [49] and Lawless and Heymann [50]. Eleven participants who consumed fish at least once every fortnight were recruited, gave consent, and were trained according to AS 2542.1.3:2014 [51] and CAC-GL 31-1999 [52]. People with allergies, smoking habits, chronic health issues, visual impairment, respiratory issues, and taste disorders, as well as those who were pregnant, breastfeeding, or on long-term medication, were excluded from the sensory evaluation to reduce the confounding factors and risks. The screening for sensory sensitivity was then conducted prior to the sensory evaluation. After both screenings, nine participants aged 18–50, consisting of 5 females and 4 males, were considered eligible and included in the study as semi-trained panelists.

In order to understand the quality of barramundi for retail display, their visual appearance, odor, and overall acceptability were first evaluated using the Labeled Magnitude Scale [53] in raw barramundi samples. After this, to investigate the cooked quality of the fish fillets, the same samples given to the panelists were sous vided at 74 °C for 6 min in vacuum-sealed boil-in pouches. “Sous vide” is a recognized cooking technique to preserve nutritional and sensory characteristics [54] and is also a recommended cooking technique for the sensory evaluation of fish (CAC-GL 1999). The samples were served to the panelists within 15 min. The cooked samples were assessed for their appearance, odor, and overall acceptability as per the raw samples but were additionally analyzed for taste and texture.

The sensory trial was conducted in strict compliance with the Australian Code for the Responsible Conduct of Research and the National Statement on Ethical Conduct in Human Research. The trial was reviewed and approved by the Curtin University Human Research Ethics Committee prior to all training and sensory evaluations conducted in this study (approval number HRE2020-0689).

### 2.5. Simulated Retail Display Protocols

The freshly filleted fish samples were unpacked and immediately placed on ice inside an uncovered polystyrene box. The box was then placed in a 4 °C refrigerator to simulate the conditions found in retail display. The melted ice was drained daily and replaced with new ice. Six fillets (2 fillets per tank) from different fishes were analysed per treatment on days 0, 4, and 8 post-slaughter.

### 2.6. Physical Parameters

#### 2.6.1. Texture Profile Analysis (TPA)

For texture, all measurements were conducted under room temperature at 24.5 °C, and the fish fillets were tempered for 30 min prior to the analyses. The fillet portion between the pelvic and anal fins of the barramundi was sampled. The flesh along the lateral line, including the dorsal and ventral portions, was then compressed using a TVT 6700 texture analyzer (PerkinElmer, Inc., Waltham, MA, USA) equipped with a 20 kg load cell and a 25 mm flat-ended cylindrical probe. Two consecutive cycles of 50% compression with 5 sec in between were conducted under a constant speed of 50 mm/min. Six texture parameters, namely the hardness, cohesiveness, adhesiveness, springiness, gumminess, and chewiness, were obtained from each analysis using Bourne’s [55] calculation methods and the TexCal 5.0 instrumental software.

#### 2.6.2. Microscopic Observation of Fillet Tissues

Portions of muscle stored at 4 °C were cut from three fillets/treatment at day 1, day 4, and day 8 and immediately fixed in 10% buffered formalin before being dehydrating with a series of alcohol washes. Then, the samples were embedded in paraffin, sectioned to 5 µm in thickness, and stained with Alcian blue for observation under a light microscope according to the standard histological procedure [18].

#### 2.6.3. Drip Loss

The fish samples were weighed daily from day 0 to day 8. The drip loss was then calculated by dividing the weight loss over the initial weight of the fresh fish sample and expressed as a percentage.

#### 2.6.4. Color

The surface color coordinates (L*, a*, b*) were obtained using HunterLab ColorFlex (Hunter Association Laboratory Inc., Reston, VA, USA) with a glass port insert. A colorimeter was calibrated prior to usage in the session. The colors were measured on both the fillet skin and flesh in the ventral and dorsal portions of the barramundi fillet along the lateral line.

#### 2.6.5. Quality Index (QI)

The QI scheme constructed by Fuentes-Amaya et al. [43] was used to determine the quality index of the barramundi fillets. In brief, the skin, appearance, and flesh of the fish were scored based on quality parameters such as the brightness, transparency, texture, blood color, odor, and gaping. The sample was then given a score out of 10 on days 1, 4, and 8.

### 2.7. Chemical Parameters

#### 2.7.1. pH

The AOAC [44] method was used to determine the pH values of the barramundi samples. First, 1 g of fish was homogenized with 10 mL of distilled water and mixed using a rotary suspension mixer (Ratek, Melbourne, VIC, Australia) for 30 min. A three-scale calibrated Aqua-pH meter (TPS Pty, Ltd., Brisbane, QLD, Australia) was then used to determine the pH values of the aliquot.

#### 2.7.2. Lipid Oxidation

The modified method used by Raharjo et al. [56] was used to determine the 2-thiobarbituric acid reactive substances (TBARs) presented in the fish fillets. First, 40 mL of 5% trichloroacetic acid (TCA) was added to 10 g of sample and homogenized along with 1 mL of 0.15% 2,6-di-teri-butil-4-methylphenol (BHT) in ethanol. The homogenized sample was then filtered and adjusted with 5% TCA to 50 mL. Next, 2 mL of the sample was transferred to a screw cap test tube and 2 mL of 0.08 M 2-thiobarbutric acid (TBA) was added. The mixture was heated at 100 °C for 10 min to allow the reaction to occur. The absorbance was then measured at 532 nm. The concentration was calculated as mg MDA kg^−1^ using standards prepared with 1,1,3,3-tetrathyoxypropane (TEP) in 20% TCA at a 1–10 µM concentration range.

### 2.8. Statistical Analysis

The tanks were considered experimental units, whereas the fish represented the sample units. The results were represented as means ± the standard error (SE). The normality and homogeneity of variance for all data from individual observations were checked using Kolmogorov–Smirnov and Levene’s tests, respectively. The results for the proximate composition and amino acid profiles were compared with the test diets with respect to the FM control using a one-way ANOVA. To determine the effects of the diet and storage time and their interaction on the fillet texture and color, a two-way ANOVA was applied with the diet and storage time as the fixed factors. If the effect was significant, a one-way ANOVA with Tukey’s post-hoc test was subsequently performed for diet and storage to determine if the means differed significantly among the test diets and storage times. The significance levels in all cases were set to * *p* < 0.05, ** *p* < 0.01, and *** *p* < 0.001.

## 3. Results

### 3.1. Proximate and Amino Acid Muscle Compositions

The moisture, protein, lipid, and ash contents of the barramundi fillets were similar in all treatments at the end of the trial (Table 2). The total essential amino acids were unaffected by the complete replacement of FM with the mixture of PBM and HI larvae meal (Table 2). However, the level of histidine was significantly lower in barramundi fed PBM-HI-based diets when compared to the 0PBM-0HI control. In barramundi fed 70PBM-30DHI, the lysine decreased significantly when compared to the 0PBM-0HI control. The summation of non-essential amino acids in the fillets of barramundi fed the PBM-HI-based diets was similar to the fillets of barramundi fed 0PBM-0HI. Among all individual non-essential amino acids, serine was negatively affected by the PBM-HI-based diets when compared to the 0PBM-0HI treatment.

### 3.2. Muscle Fatty Acid Composition

The influence of the PBM-HI-based diets on the individual fatty acid and total fatty acid concentrations in the muscle of barramundi is presented in Table 3. The PBM-HI-based diets elevated the concentration of total saturated fatty acids (SFA) due to an increase in the concentrations of C12:0 and C14:0. Similarly, an upsurge in C16:1 n7 and C18:1 cis + trans increased the amount of total monounsaturated fatty acids (MUFAs) in the PBM-HI-based diets. Meanwhile, the lower levels of C20:5 n3, C22:6 n3, C22:5 n3, and C22:4 n6 resulted in a decrease in polyunsaturated fatty acids (PUFAs), n-3 PUFAs, n-6 PUFAs, and the ratio of n-3 to n-6 PUFAs in the experimental diets.

### 3.3. Sensory Evaluation

The fillet sensory attributes of barramundi fed the mixture of PBM and HI larvae meal compared to the FM control diet are presented in Table 4. The raw quality of the barramundi fillets was improved in the PBM-HI-based diet, as indicated by the high numbers of merit points given by the panelists for their appearance, odor, and overall quality. A similar trend was observed after cooking for all attributes, but only the cooked odor improved significantly in the fillets of the barramundi fed PBM-HI-based diets when compared to the fillets of the barramundi fed the FM control diet.

### 3.4. Microstructure and Texture Profiles of Fillets

**Figure 1:**
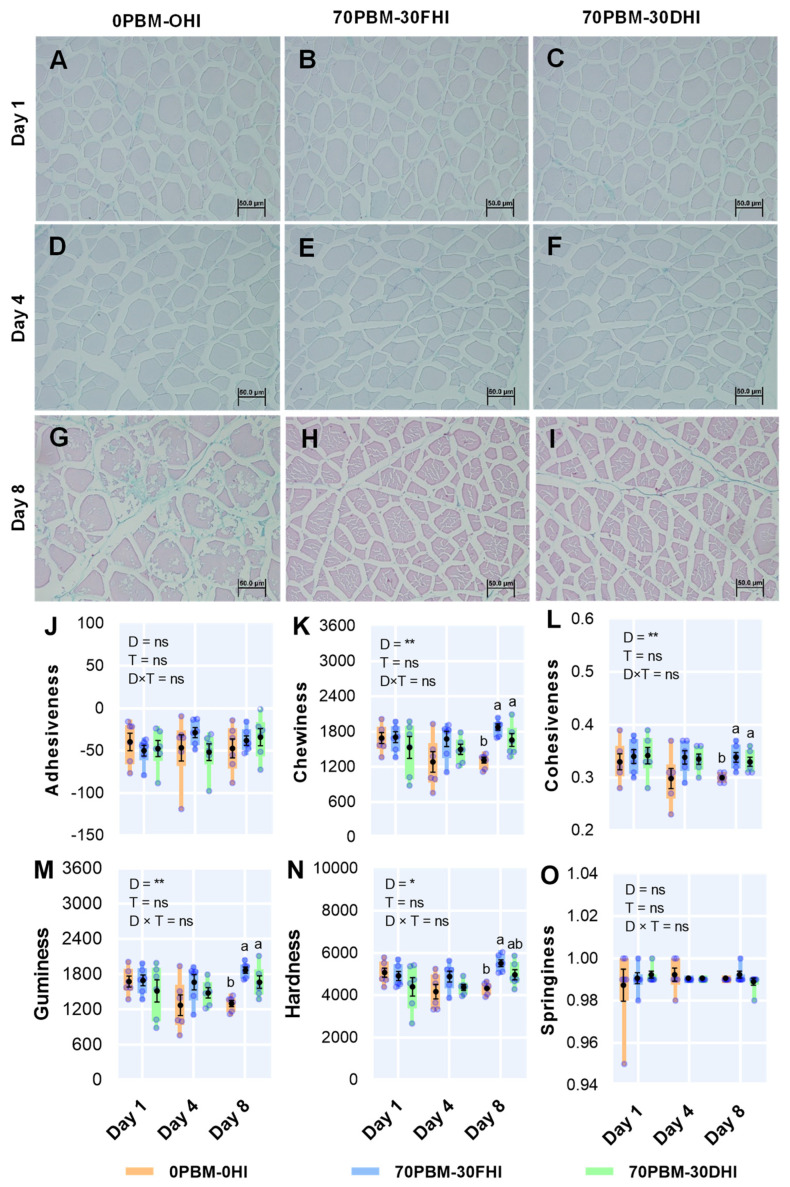
Representative muscle histology results at day 1 (**A**–**C**), day 4 (**D**–**F**), and day 8 (**G**–**I**) of fillets being stored at 4 °C and changes in the fillet texture profiles, including adhesiveness (**J**), chewiness (**K**), cohesiveness (**L**), gumminess (**M**), hardness (**N**), and springiness (**O**), for barramundi fed complete replacement of fishmeal (FM) diets with mixtures of poultry by-product meal (PBM), full-fat *Hermetia illucens* (FHI), and partially defatted *Hermetia illucens* (DHI) larvae meal. The semi-transparent light blue round markers indicate individual datapoints (*n* = 6), while the box–whisker plot of individual datapoints and black marker denote the mean and standard error values, respectively. Lower cases in the figure indicate significant differences between the treatments. The asterisks represent the significant differences at * *p* < 0.05, and ** *p* < 0.01; ns denotes non-significant.

The changes in the microstructure of the muscle tissues and the texture profile, including the adhesiveness, chewiness, cohesiveness, gumminess, hardness, and springiness, are presented in Figure 1. The muscle structures at days 1 (Figure 1A–C) and 4 (Figure 1D–F) were unchanged in the fillets of barramundi fed any of the test diets, as manifested by the tight attachment and regular shape of the myofibrils together with the uniform distribution and distinct connective tissues. However, at day 8, severe muscle degeneration and atrophy in cells of 0PBM-0HI-fed fish was observed (Figure 1G), while the PBM-HI-based diets prevented such structural changes (Figure 1H,I). The adhesiveness (Figure 1J) and springiness (Figure 1O) were not changed by the diet or storage time, while the diets significantly changed the chewiness (Figure 1K), cohesiveness (Figure 1L), gumminess (Figure 1M), and hardness (Figure 1N), which were higher in the fillets of barramundi fed the mixtures of PBM, FHI, and DHI larvae meal. However, the storage time did not influence those parameters. There was no interaction between “diet” and “storage time” in any of the texture profiles.

### 3.5. Drip Loss

**Figure 2:**
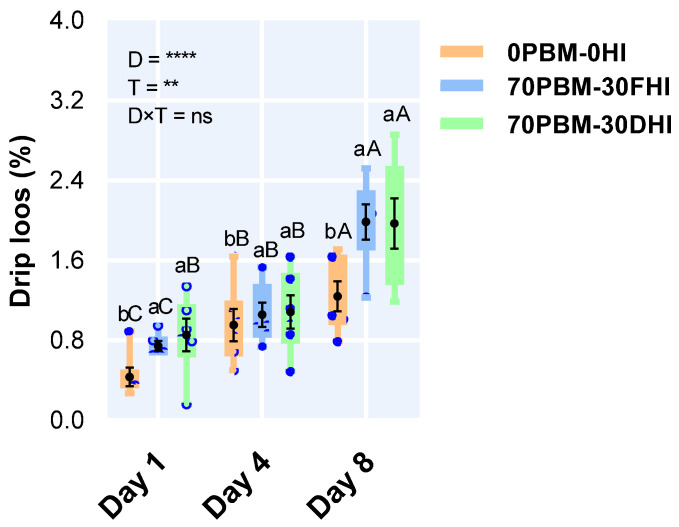
Changes in drip loss after the 8-weeks feedin trial and at day 1, day 4, and day 8 when stored at 4 °C in barramundi fed complete replacement of fishmeal (FM) diets with a mixture of poultry by-product meal (PBM), full-fat *Hermetia illucens* (FHI), and partially defatted *Hermetia illucens* (DHI) larvae meal. Semi-transparent light blue round markers indicate individual datapoints (*n* = 6), while the box–whisker plot of individual datapoints and black markers denote the mean and standard error values, respectively. Lower case letters indicate significant differences between the treatments, while upper cases in the figure indicate significant differences between the storage days. Asterisks represent the significant differences at ** *p* < 0.01, and **** *p* < 0.001; ns denotes non-significant.

The drip loss was affected by the type of diet, with a significantly higher drip loss rate in the fillets of barramundi fed the PBM-HI-based diets during the storage time (Figure 2). The drip loss increased over the storage time in the fillets of all barramundi fed test diets. No interaction was observed between diet and storage time.

### 3.6. Skin Color

**Figure 3:**
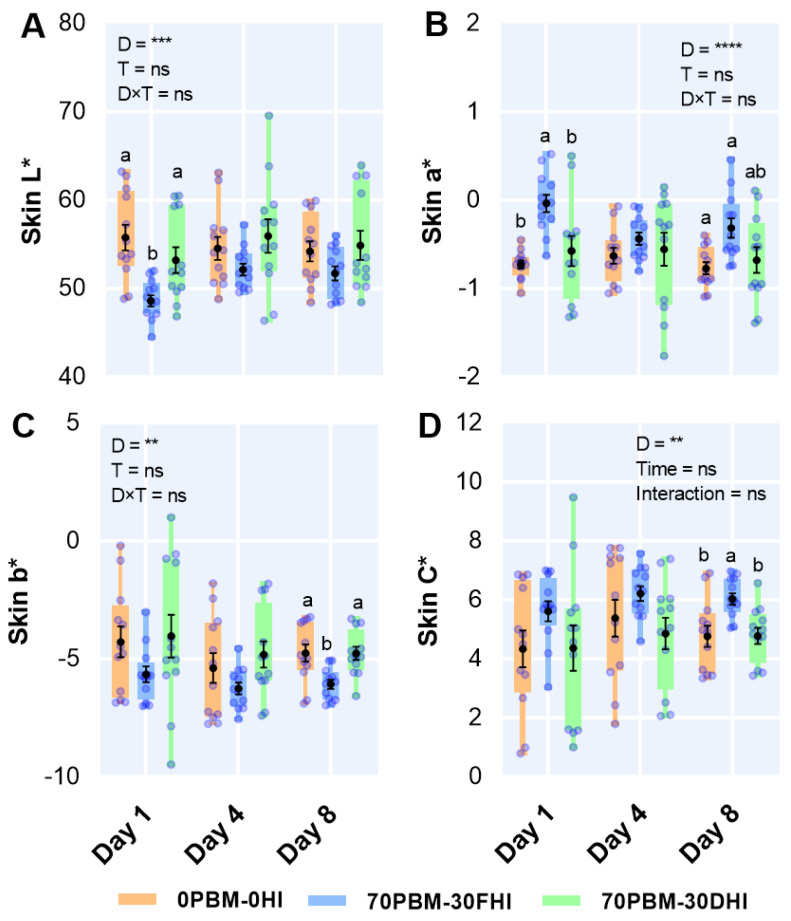
Changes in the fillet skin color, including the lightness (L*) (**A**), redness (a*) (**B**), yellowness (b*) (**C**), and chroma (**D**) levels, after the 8-week feeding trial and at day 1, day 4, and day 8 when stored at 4 °C in barramundi fed a complete replacement of fishmeal (FM) diet with mixtures of poultry by-product meal (PBM), full-fat *Hermetia illucens* (FHI), and partially defatted *Hermetia illucens* (DHI) larvae meal. Semi-transparent light blue round markers indicate individual datapoints (*n* = 12), while the box–whisker plot of individual datapoints and black markers denote the mean and standard error values, respectively. Lower cases indicate significant differences between the treatments. Asterisks represent the significant differences at ** *p* < 0.01, *** *p* < 0.001 and **** *p* < 0.0001 and ns denotes non-significant.

The two-way ANOVA analysis showed that the skin colors in terms of the L* (brightness), a* (redness), yellowness (b*), and chroma levels were significantly influenced by the diet. The L* level on day 1 decreased significantly in the skin of barramundi fed 70PBM-30FHI when compared to the skin of barramundi fed 0PBM-0HI and 70PBM-30DHI. Meanwhile, the skin L* level (Figure 3A) was unchanged on days 4 and 8 when fed either 0PBM-0HI or test diets. The skin a* level (Figure 3B) was higher in barramundi fed 70PBM-30FHI and 70PBM-30DHI on days 1 and 8, with no variation on day 4 between the diets. On day 8, the skin b* level (Figure 3C) decreased in barramundi fed 70PBM-30FHI, while the skin chroma level (Figure 3D) improved in fish fed 70PBM-30FHI when compared to the control. However, there was no variation in skin b* or chroma levels between the test diets on days 1 and 4. The storage time had no influence on the skin color, with no interactive effects.

### 3.7. Flesh Color

**Figure 4:**
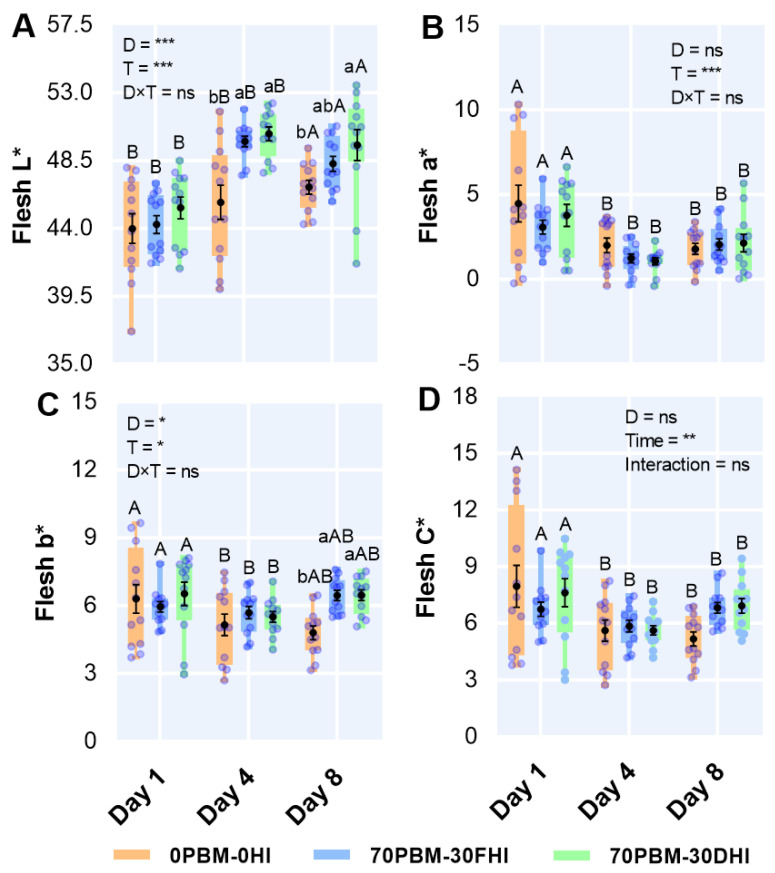
Changes in the fillet flesh color, including the lightness (L*) (**A**), redness (a*) (**B**), yellowness (b*) (**C**), and chroma (C*) (**D**) levels, after the 8-week feeding trial and at day 1, day 4 and day 8 when stored at 4 °C in barramundi fed complete replacement of fishmeal (FM) diets with mixtures of poultry by-product meal (PBM), full-fat *Hermetia illucens* (FHI), and partially defatted *Hermetia illucens* (DHI) larvae meal. Semi-transparent light blue round markers indicate individual datapoints (*n* = 12), while the box–whisker plot of individual datapoints and black markers denote the mean and standard error values, respectively. Lower cases indicate significant differences between the treatments, while upper cases in the figure indicate significant differences between the storage days. Asterisks represent the significant differences at * *p* < 0.05, ** *p* < 0.01, and *** *p* < 0.001 and ns denotes non-significant.

The fillet flesh color results in response to the test diets and storage time are presented in Figure 4. The two-way ANOVA showed that the diet type had a significant effect on the flesh L* and b* (Figure 4A,B); however, the storage time influenced all color attributes. On days 4 and 8, the flesh brightness improved significantly in PBM-HI-fed barramundi fillets (Figure 4A), but the flesh yellowness (Figure 4C) increased only in the barramundi fed PBM-HI-based diets on day 8. Irrespective of the diet type, the flesh lightness in all test diets increased with time, while the redness (Figure 4A), yellowness (Figure 4B), and chroma (Figure 4D) levels decreased over the storage time.

### 3.8. Quality Index (QI) Method

**Figure 5:**
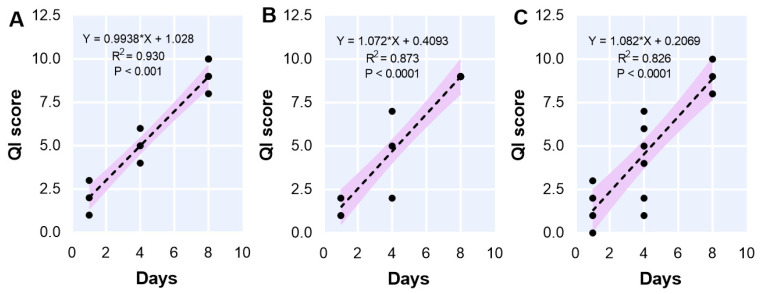
Correlations between the quality index (QI) values and storage times for the test diets, including 0PBM-0HI (**A**), 70PBM-30FHI (**B**), and 70PBM-30DHI (**C**).

The changes in fillet quality as determined by the barramundi fillet QI scheme developed by Fuentes-Amaya et al. [43] in response to diets and storage times are presented in Table 5. The diet type had no significant effect on the QI value, while the storage time influenced the QI attributes. There was no interaction between the diet and storage time. The QI increased linearly with the increasing storage times (Figure 5), with maximum demerit points of 10 for 0PBM-0HI (Figure 5A), 9 for 70PBM-30FHI (Figure 5B), and 10 for 70PBM-30DHI (Figure 5C), respectively.

### 3.9. pH and Lipid Oxidation

**Figure 6:**
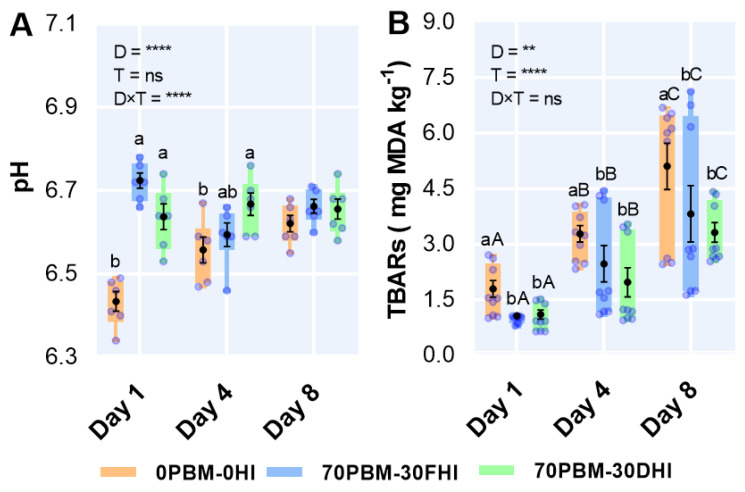
Changes in the fillet flesh pH (**A**) and TBARs (**B**) values after the 8-week feeding trial and at day 1, day 4, and day 8 when stored at 4 °C in barramundi fed complete replacement of fishmeal (FM) diets with mixtures of poultry by-product meal (PBM), full-fat *Hermetia illucens* (FHI), and partially defatted *Hermetia illucens* (DHI) larvae meal. Semi-transparent light blue round markers indicate individual datapoints (*n* = 9), while the box–whisker plot of individual datapoints and black markers denote the mean and standard error values, respectively. Lower cases indicate significant differences between the treatments, while upper cases in the figure indicate significant differences between the storage days. Asterisks represent the significant differences at ** *p* < 0.01, and **** *p* < 0.0001 and ns denotes non-significant.

The pH and lipid oxidation levels, as measured by using malondialdehyde (MDA), in response to the test diets and storage time are presented in Figure 6. The two-way ANOVA demonstrated that the pH was influenced by the test diets, with a significant interaction between the diet type and storage time (Figure 6A). The pH increased significantly in the flesh of barramundi fed PBM-HI-based diets at days 1 and 4 compared with the control-fed barramundi. However, the storage time did not influence the pH level. The rancidity test result as evaluated by the TBAR activity levels at all storage times was lower in the flesh of PBM-HI-fed barramundi than in the control fish (Figure 6B). However, the TBAR activity levels increased in all test diets with storage time.

## 4. Discussion

In evaluating the use of alternate processed animal protein sources such as PBM and HI larvae meal in aquafeed, as well as in the production analyses, it is important to also investigate if the physicochemical, sensory, and shelf-life characteristics of the aligned edible products will be altered by the alternative diets. The present study used fillets derived from a 56-day feeding trial testing a mixture of HI larvae meal and PBM diets compared with an FM control diet. The fillets were evaluated for a range of nutritional, sensory, and shelf-life attributes (in the simulated retail display) from the different dietary treatments.

The retention of protein and essential amino acids, one of the most sensitive indicators of fish health, has been used to evaluate the adequacy of the supply of amino acids in the dietary composition [57,58]. Feeding with 75% of PBM only or bioprocessed PBM for 42 days negatively impacted most of the essential and non-essential amino acids in the muscle tissue of barramundi [59]. Similarly, replacing FM with 60% PBM protein reduced the retention of total EAAs, particularly lysine and methionine, in juvenile black sea bream, *Acanthoparus schlegelii* [60], while the replacement of FM with 50 and 100% of PBM negatively impacted the threonine levels in the fillets of juvenile gilthead seabream, *Sparus aurata* [61]. The muscle amino acid profile of the fish in the present study was unaffected by the inclusion of PBM-HI in the barramundi diets compared with a standard FM diet. Equally as important, the fillet food quality parameters were not decreased by the change in diet.

The amino acid profile in PBM-HI-fed barramundi was similar to those reported for juvenile gilthead seabream, *Sparus aurata*, fed 50 and 100% PBM [61], and juvenile spotted rose snapper, *Lutjanus guttatus,* fed PBM (up to 75%) [62]. The retention of amino acids in fish muscles when fed PBM may have been improved in the current study by the addition of HI larvae meal, which contains low molecular weight peptides, mainly ranging between 20 and 245 kDa, providing more free amino acids [63]. Additionally, it has been reported that HI larvae protein contains 6% free amino acids (<1000 Da) as compared to 2.2% (of total proteins) in FM [64]. Free amino acids reach the intestine and are absorbed by enterocytes faster than the intact proteins [19,65,66], which may explain the beneficial effect of incorporating HI larvae protein to prevent the PBM-only-induced negative impact on the barramundi muscle AA composition, as previously reported [67].

The inclusion of PBM alone decreased the sensory quality of female tenches, *Tinca tinca* [68], while meat meal inclusion did not affect the sensory quality of barramundi [11]. In our experiments, the highest score given by the panelists to the raw and sous-vide-cooked fillets of barramundi fed PBM-HI suggests that feeding with HI larvae containing chitin and other functional molecules [67] might lead to improved fillet quality. Chitosan, the deacetylated form of chitin coatings, has been reported to improve the sensory quality of seafood [69,70,71], while the fillets of salmon fed a complete replacement of FM diet with HI larvae meal were evenly appreciated with the control diet, except for small decreases in color intensity and textural attributes [72]. The highly positive perceptions of the panelists to the PBM-HI-based fillets reported here strongly indicate that consumers will accept the replacement of FM with this feed base in barramundi diets. It is also noteworthy that the fillet sensory quality in our study was consistent with the instrumental texture and lipid oxidation results.

Consumers generally distinguish texture attributes such as firmness and elasticity in fish [73,74] associated with adhesiveness, chewiness, cohesiveness, gumminess, hardness, and springiness, all of which are broadly used to evaluate the meat quality of the fish [33,75,76,77]. Amongst the external and internal factors, the diet is considered one of the factors directly influencing the fillet texture [78,79,80,81] and tissue structure [7]. Previous shelf-life studies have indicated that bacterial protease acts on the muscle protein and connective tissues post-mortem, leading to muscle disintegration in fish [82,83,84]. The alteration of the muscle tissue structure during storage has been shown to be associated with changes in texture profiles, especially decreases in the hardness, cohesiveness, gumminess, and chewiness [85,86]. Hence, the improvement in the flesh texture in our study as assessed by the chewiness, cohesiveness, gumminess, and hardness of barramundi fed PBM-HI at day 8 may indicate the ability of functional components of HI larvae to slow the degradation process and preserve the texture in comparison to the control diet. This aligns with the histological results obtained from the microstructure observations. As mentioned earlier, the HI larvae protein containing low molecular weight peptides was able to protect against cellular damage resulting from neutrophil and myeloperoxidase responses [64]. There is a lack of information regarding the influence of HI larvae meal alone or in a mixture with PBM on the texture profile of barramundi to allow a comparison with the present study. However, feeding 2.5, 5, and 7.5% with HI larvae meal for 10 weeks improved the muscle texture in eels *(Monopterus albus*) [87]. In contrast, feeding with the mealworm, *Tenebrio molitor,* for 113 days did not influence the hardness, cohesiveness, gumminess, or adhesiveness of the fillet muscles of sea bream, *Pagellus bogaraveo* [31]. Chitosan, a derivative of chitin, has been reported to have antioxidant [88] and antimicrobial properties and is widely used as a preservative agent in food applications [89,90,91,92]. Chitosan has successfully been transformed from the chitin from HI larvae meal [93], but the dietary application of insect-based chitosan for improving the muscle texture of fish has not yet been studied. However, the underlying hypothesis behind the antimicrobial mechanism is the interaction between the positively charged NH3+ group in chitosan and the negatively charged microbial cell membranes, which induces a change in the bacterial cell permeability [91,94,95,96]. Such a mechanism might have helped in improving the muscle texture in fillets of barramundi fed a PBM-HI-based diet until day 8.

In addition to the above-mentioned functional molecules, lauric acid (C12:0), a predominant medium-chain fatty acid in HI larvae, also has antioxidant and antimicrobial properties [97,98]. A significant increase in the concentration of C12:0 in the muscles of barramundi fed PBM-HI might have slowed the oxidation process in the muscles, thereby influencing the texture quality. The chemical composition of the fish muscles, particularly in thers of the proteins and lipids, had a major influence on the flesh texture quality of sea bass, but no differences in the lipid and protein contents were found among the experimental groups. The PBM-HI-based diets showed higher contents of glutamic acid and arginine, which have been reported to improve the texture by increasing the muscle cell density and connective tissue [99,100]. Further research should be conducted to determine whether the chitin, lauric acid, choline, polysaccharides, and antimicrobial peptides in HI larvae meal influence the flesh texture of barramundi.

The drip loss is an important quality parameter associated with the juiciness, appearance, and color of the fish product [101], as well as directly affecting the weight of the fillets (thereby determining the economic revenue). On all days, the fillets of barramundi fed PBM-HI-based diets showed higher drip loss rates (0.74–1.98%) than the fillets of barramundi fed the FM control diet (0.43–1.24%). Nevertheless, the weight changes were low compared to other studies. The percentage of drip loss reached 3% for rested and conventionally harvested barramundi fillets at 4 days post-thawing in another study [38]. Additionally, the drip loss was around 35% in barramundi fillets treated with high pressures and a chitosan coating during 8 days of chilled storage at 4 ± 1 °C [102]. The drip loss in meat has been shown to be highly related to lipid oxidation, as peroxidation can cause severe damage to membrane proteins, thereby lowering the pH [103,104]. However, the TBAR and pH results in this study do not align with this hypothesis, as the fish fed PBM-HI were shown to have lower lipid oxidation and normal pH levels. Such changes in drip loss could be related to the different types of histological damage shown in Figure 1G–I. In test diets, an increase in intrafibrillar cavities while maintaining the integrity of sarcolemma was reported, while the control was shown to have severe sarcolemma damage with no evidence of an increase in spaces between myofibrilla [105]. In terms of the water withheld in the muscle cells, 85% is estimated to be held in the myofibrilla, and it has been hypothesized that the gradual mobilization of water from the intramyofibrillar space is the key source of drip loss [105]. An increase in space between the myofibrilla could be caused by sarcoplasmic fluid forced from the myofilament spacing, thereby elevating the drip loss. Further studies of the muscle microstructure with insect-fed diets are needed to validate the current findings. Regardless of the diet type, the drip loss increased continuously with storage time in all test diets, with the results being consistent with the lipid oxidation changes observed over the storage time. In short, the drip loss rates ranging from 0.43 to 1.98% in this study cannot be considered high; therefore, they are not a major problem in chilled barramundi, as supported by many earlier studies [106,107,108].

The color is considered one of the important attributes in assessing the fillet quality of fish, often being in direct association with the acceptance or rejection of a product by the consumer. Farmed barramundi often look greyer than wild barramundi, directly impacting consumer acceptance and forcing farmed barramundi into a lower market position. An attempt to manipulate the melanin synthesis pathway by adding some precursors such as tyrosine, tryptophan, and tyrosinase into the diets that were fed to he barramundi for six weeks to prevent greyish-blue coloration did not resolve the discoloration [109]. Both the skin and muscle lightness were significantly influenced by the diet and storage time in the present study. The underlying reasons behind the improvement in the brightness (L*) of the barramundi fillets from fish fed PBM-HI-based diets could be related to reductions in melanosis, which has been linked to the greying of farmed fish, as reported by Howieson, Glencross, Little, Bourne, Aris, Partridge, Paton, Tonkin, Allan, Wilkinson, and Smullen [109]; Cooper and Midling [110]; and Cooper et al. [111]. Aside from melanosis, the mechanisms associated with greying have not been well studied, and the exact biochemical pathways are unknown. Valente et al. [112] reported that different rearing conditions affected the brightness and skin color of gilthead sea bream. However, the rearing conditions of the barramundi in the present study were uniform, meaning the dietary intervention may be the primary determinant of color differences.

The gradual increase in flesh color saturation we noted with storage was similar to that reported by Jones and Carton [37]. Since fish do not have the de novo power to synthesize carotenoids, the presence of carotenoids in the diet can influence the fish color. Generally, insects are a good source of β-carotene [113], which influenced the redness index (a*) of blackspot seabream, *Pagellus bogaraveo,* fed 50% mealworm, *Tenebrio molitor* [114]. The same effects were observed in the skin of the HI-larvae-based diets in the present study, raising the possibility that the barramundi utilized β-carotene from the HI larvae inclusion. The unchanged flesh redness in the barramundi fed PBM-HI-based diets was similar to the muscle redness of European seabass, *Dicentrarchus labrax* [22], and rainbow trout [115] fed HI larvae and prepupae-meal-based diets. Regardless of the diets, the decrease in the fillet redness over the storage time in our test diets was consistent with the findings of Jones and Carton [37], who reported progressive color changes in the fillets of barramundi subjected to two weeks storage at 2 °C, irrespective of feeding with commercial diets supplemented with α-tocopherol acetate for five months.

Furthermore, most edible insects contain riboflavin (vitamin B2), a yellow-colored pigment whose quantity is extremely variable [31] but may influence the skin and flesh colors of fish fed insect-based diets. For instance, feeding with 25 and 50% of mealworm for 131 days increased the flesh yellowness of blackspot sea bream, *Pagellus bogaraveo* [31]. HI larvae meal has been reported to contain 2.2 mg/100 g riboflavin [116], which could have caused the increased yellowness in the skin of the fish fed 70PBM-30DHI and the flesh of the fish fed insect-based PBM diets in the present study. However, in our previous study, feeding with PBM-based diets supplemented with 5 and 10% of HI larvae and tuna hydrolysate for six weeks did not influence the skin or flesh color of barramundi [19]. A similar influence on the yellowness of juvenile fillets of European seabass, *Dicentrarchus labrax,* was found when fed pre-pupae larvae [22].

A strong correlation was observed between the QI and storage time values for the 0PBM-0HI (R2 = 0.93), 70PBM-30FHI (R2 = 0.87), and 70PBM-30DHI (R2 = 0.83) diets, indicating that the freshness gradually deteriorated with time. The control treatment degraded the fastest, which was aligned with the texture microstructure results. The increase in QI values over time was consistent with the previous report on the QI values of vacuum-packaged barramundi fillets stored at 4 °C [43]. The correlation between the QI and storage time was expected, since a similar correlation has been reported by others [117,118,119,120,121,122]. The maximum demerit score for 0PBM-0HI (10) followed by 70PBM-30FHI (9) and 70PBM-30DHI (9) at day 8 was consistent with lipid oxidation and the microstructure of the muscle tissues. The demerit score at day 4 for all test diets was below the previously reported QI score (7.5) for vacuum-packaged barramundi fillet stored at 4 °C [43].

It has been reported that reductions in fish muscle pH are associated with color degradation, muscle gaping, blood spotting, flesh texture alterations, and drip loss [37,38,123]. The increase in pH in the flesh of the barramundi fed PBM-HI was similar to the changes in fillet pH reported after slaughtering and three days of storage in European seabass fed HI pre-pupae larvae meal [22]. Furhtermore, pH increases in fish fillets have been reported to be associated with the production of alkaline compounds such as ammonia and biogenic amines as a result of either bacterial activity or endogenous enzyme activity [124]. Alternatively, pH decreases have been linked to the production of lactic acid caused by the anaerobic fermentation of glycogen and the liberation of inorganic phosphates from ATP degradation at the rigor stage [125,126]. It is noteworthy that the pH range in the present study, irrespective of the diet and storage time effects, indicated no production of non-desirable acidic or alkaline compounds, suggesting that PBM-HI-based diets can delay the spoilage process in barramundi muscle. Further, the pH range was within the range reported for fresh barramundi flesh [38]. The increases in pH at certain levels have been reported to prevent the production of volatile bases, since bacteria can take energy from oxidative products resulting from lipid oxidation rather than glycogen and other normal substrates [37]. Hence, there was an inverse correlation reported between the pH and degree of lipid oxidation in the flesh of barramundi over a storage period of 14 days [37]. Similarly, the pH of the flesh in the present study appeared to be inversely proportional to the production of TBARs. TBARs, a measure of the carbonyl-like MDA concentration, can be formed as primary and secondary oxidative products from the degradation of polyunsaturated fatty acids, consequently generating an unpleasant taste and rancid flavor in fatty and protein-rich foods [127,128]. The fatty acid results in our previous study demonstrated that fish fed with PBM-HI have less PUFAs [44], making them prone to oxidation due to the lower energy requirement to remove the hydrogen from unstable carbon double bonds [129]. Lower production rates of MDA could, therefore, be related to a lower PUFA content or higher content of saturated fatty acids in the muscle, particularly lauric acid, which is characterized by its potential antioxidant properties.

A previous study on European seabass [22] also suggested that the antioxidant properties of HI could positively improve the free radical scavenging ability of fish, leading to the inhibition of lipid oxidation. This assumption was proven by Mouithys-Mickalad, Schmitt, Dalim, Franck, Tome, van Spankeren, Serteyn, and Paul [64], who observed strong antioxidant potential in HI larvae protein by evaluating the DPPH radical scavenging activity. The lower production rate of MDA in the present study might be attributed to the presence of chitin based on the in vitro and in vivo free radical scavenging activity levels, as well as the lauric acid in the HI larvae meal. In addition, several studies have already reported on the antioxidant activity levels of chitosan and the deacetylated form of chitin, as well as the reduced lipid oxidation rates in fish and fish products [69,70,130,131,132]. The increase in TBAR production in the fillets of barramundi fed test diets over the storage time was similar to a study by Moutinho, Pedrosa, Magalhães, Oliva-Teles, Parisi, and Peres [22], who found a similar increase in MDA production with increasing storage time in the fillets of European seabass when fed different levels of HI pre-pupae larvae meal. However, the MDA production levels in the present study were below the reported critical limits (7–8 mg MDA/kg) [133,134]. The results for the pH and TBAR values in the barramundi fillets might be associated with the microbial activity; hence, further studies are needed to evaluate the whole microbial profile in barramundi fillets using a modern tool to investigate whether HI larvae can delay the production of spoilage bacteria.

## 5. Conclusions

The ongoing aquaculture nutrition research investigating sustainable aquafeed ingredients to replace FM, focusing on the growth and physiological parameters of the cultured fish, sometimes lacks rigorous food science characterization information, including nutritional data, as well as sensory and shelf-life considerations for the aligned edible fillet products. Such edible product quality information is important to ensure the market acceptance of the product, as well as ensure the inherent fillet characteristics are not impacted. In this study, we were able to demonstrate that the inclusion of HI larvae meal, either full-fat or defatted, in combination with PBM was effective in replacing FM entirely in barramundi diets, without changing the fillets’ proximate or amino acid composition. The PBM-HI-based diets also improved the sensory quality of the barramundi fillets relative to those from FM-fed fish. The PBM-HI-based diets were also shown to be effective in improving the fillet brightness and texture and in delaying the damage to the sarcolemma and lipid oxidation during chilled storage. These results strongly support the inclusion of PBM-HI to replace 100% FM protein in aquafeed to enhance the farmed barramundi fillet quality and prevent rancidity during storage. Further, such changes in the feed base could enhance the consumer acceptability of alternative protein ingredients, thereby supporting the development of a circular bioeconomy in aquafeed production. The new diet may also resolve the “blue-greyish” coloration problem identified in farmed barramundi. Further long-term fish feeding trials with larger fish fillets, including microbiological assessments and investigations of the sensory impacts of different cooking methods, would be instructive to fully understand the beneficial effects of the inclusion of HI larvae and PBM on the final product quality of barramundi.

## Figures and Tables

**Table 1:** The nutrient contents of the test diets and different ingredients such as poultry by-product meal (PBM), full-fat *Hermetia illucens* (FHI), and partially defatted *Hermetia illucens* (DHI). The ingredients and proximate compositions were previously published in our earlier article [15].

Ingredients (g/100 g)	0PBM-0HI	70PBM-30HI	70PBM-30DHI	PBM *	FHI *	DHI
FM	72.00	0.00	0.00	-	-	-
PBM	0.00	50.50	50.50	-	-	-
Canola oil	1.00	0.50	0.50	-	-	-
Full-fat HI	0.00	35.00	0.00	-	-	-
Defatted HI	0.00	0.00	27.83	-	-	-
Corn/wheat starch	7.00	5.90	11.00	-	-	-
Lecithin-Soy (70%)	1.00	2.00	1.00	-	-	-
Vitamin C	0.05	0.05	0.05	-	-	-
Dicalcium Phosphate	0.05	0.05	0.05	-	-	-
Wheat (10 CP)	16.90	4.00	7.07	-	-	-
Vitamin and mineral premix	0.50	0.50	0.50	-	-	-
Salt (NaCl)	1.00	1.00	1.00	-	-	-
Cod liver oil	0.50	0.50	0.50	-	-	-
Nutritional Composition (%)						
Dry matter	89.95	90.21	90.16	-	-	-
Crude protein	47.88	47.94	48.06	-	-	-
Crude lipid	12.59	13.61	13.96	-	-	-
Ash	11.23	11.69	11.51	-	-	-
Essential amino acid (% of total amino acid)						
Arginine	6.40	6.66	6.64	7.32	5.45	5.31
Histidine	3.24	2.52	2.52	2.96	3.27	3.17
Threonine	4.71	4.16	4.18	4.19	4.35	4.39
Lysine	7.50	6.64	6.57	6.58	7.10	6.78
Methionine	2.88	2.10	2.07	2.23	2.02	1.97
Valine	5.64	5.63	5.62	4.92	6.40	6.70
Isoleucine	4.91	4.51	4.47	4.07	4.86	5.03
Leucine	8.16	7.52	7.44	7.37	7.59	7.63
Phenylalanine	4.55	4.36	4.33	4.09	4.67	4.88
Non-Essential amino acid (% of total amino acid)						
Serine	4.39	4.27	4.30	4.34	4.48	4.43
Glycine	7.96	9.62	9.79	10.13	5.86	6.01
Aspartic acid	9.57	9.41	9.21	8.50	10.42	10.93
Glutamic acid	14.42	14.73	14.94	13.83	13.42	13.46
Alanine	7.10	7.28	7.36	6.61	6.59	7.06
Proline	5.65	6.87	7.01	6.63	6.24	6.22
Tyrosine	2.92	3.71	3.55	2.97	5.97	6.05

* The amino acid compositions of PBM and full-fat HI larvae meal were published in our earlier study [19].

**Table 2:** The proximate composition and amino acid profiles of skinless whole fillets of barramundi fed with a mixture of poultry by-product meal (PBM), full-fat *Hermetia illucens* (FHI), and partially defatted *Hermetia illucens* (DHI) meal for the complete replacement of FM. Values are means of six biological replicates ± the standard error (SE). Means with different superscript letters within the same row indicate significant differences, while means showing no superscript letters indicate no variation among the treatments. Means were compared using a one-way ANOVA with Tukey’s post-hoc multiple comparisons test at *p* < 0.05.

	0PBM-0HI	70PBM-30HI	70PBM-30DHI	*p*-Value
Proximate composition (%, Wet basis)				
Moisture	76.10 ± 0.25	76.71 ± 0.42	76.70 ± 0.41	0.46
Crude protein	20.58 ± 0.56	19.82 ± 0.03	20.33 ± 0.30	0.38
Crude lipid	1.63 ± 0.15	1.90 ± 0.30	1.69 ± 0.36	0.79
Ash	1.10 ± 0.07	1.10 ± 0.02	1.19 ± 0.03	0.35
Essential amino acids (% of total amino acids)				
Arginine	6.30 ± 0.02	6.32 ± 0.03	6.33 ± 0.03	0.48
Histidine	2.40 ± 0.04 ^a^	2.28 ± 0.02 ^b^	2.28 ± 0.02 ^b^	0.00
Threonine	4.65 ± 0.01	4.61 ± 0.03	4.61 ± 0.02	0.11
Lysine	9.71 ± 0.02 ^a^	9.71 ± 0.06 ^a^	9.49 ± 0.13 ^b^	0.03
Methionine	3.22 ± 0.03	3.19 ± 0.03	3.17 ± 0.02	0.08
Valine	5.37 ± 0.02	5.31 ± 0.02	5.34 ± 0.02	0.19
Isoleucine	5.12 ± 0.03	5.06 ± 0.04	5.06 ± 0.06	0.25
Leucine	8.49 ± 0.03	8.46 ± 0.04	8.43 ± 0.06	0.68
Phenylalanine	4.62 ± 0.03	4.64 ± 0.02	4.64 ± 0.03	0.79
Non-essential amino acids (% of total amino acids)				
Serine	4.28 ± 0.07 ^a^	4.15 ± 0.03 ^b^	4.13 ± 0.02 ^b^	0.01
Glycine	6.12 ± 0.15	6.22 ± 0.30	6.46 ± 0.21	0.24
Aspartic acid	10.44 ± 0.08	10.55 ± 0.05	10.52 ± 0.17	0.45
Glutamic acid	15.97 ± 0.07	16.12 ± 0.08	15.98 ± 0.10	0.14
Alanine	6.40 ± 0.05	6.45 ± 0.07	6.52 ± 0.06	0.09
Proline	3.58 ± 0.05	3.60 ± 0.10	3.72 ± 0.08	0.15
Tyrosine	3.35 ± 0.01	3.33 ± 0.06	3.33 ± 0.02	0.80

**Table 3:** The fatty acid composition of barramundi fillets (skinless) from fish fed with a mixture of poultry by-product meal (PBM), full-fat *Hermetia illucens* (FHI), and partially defatted *Hermetia illucens* (DHI) larvae meal for 56 days for the complete replacement of FM, obtained from our earlier study [15]. The results are expressed as means ± the standard error (*n* = 3) and different superscript letters within the row represent significant variation between the treatments. ∑ indicates the summation of individual fatty acids.

	0PBM-0HI	70PBM-30FHI	70PBM-30DHI	*p*-Value
C12:0	1.39 ± 0.85 ^c^	10.29 ± 0.15 ^a^	6.52 ± 0.35 ^b^	0.00
C14:0	2.30 ± 0.15 ^c^	3.76 ± 0.03 ^a^	3.00 ± 0.15 ^b^	0.00
C16:0	19.26 ± 0.24 ^a^	18.09 ± 0.07 ^b^	18.33 ± 0.18 ^b^	0.01
C16:1 n7	3.35 ± 0.15 ^b^	4.17 ± 0.03 ^a^	4.13 ± 0.17 ^a^	0.01
C18:1 cis+trans	25.53 ± 1.56 ^b^	31.01 ± 0.06 ^a^	34.57 ± 0.63 ^a^	0.00
C18:2 cis	9.59 ± 0.80 ^b^	14.75 ± 0.07 ^a^	14.40 ± 0.25 ^a^	0.00
C18:3 n6	0.42 ± 0.09 ^b^	0.76 ± 0.03 ^a^	0.99 ± 0.03 ^a^	0.00
C18:3 n3	2.00 ± 0.10 ^b^	2.90 ± 0.00 ^a^	2.69 ± 0.06 ^a^	0.00
C18:4 n3	0.41 ± 0.00 ^b^	0.58 ± 0.00 ^a^	0.49 ± 0.03 ^b^	0.00
C20:3 n6	0.36 ± 0.03 ^b^	0.42 ± 0.00 ^b^	0.66 ± 0.09 ^a^	0.01
C20:4 n6	1.93 ± 0.07 ^a^	1.47 ± 0.03 ^a^	1.57 ± 0.23 ^a^	0.00
C20:5 n3	2.51 ± 0.20 ^a^	1.37 ± 0.00 ^b^	1.31 ± 0.10 ^b^	0.00
C22:4 n6	1.58 ± 0.25 ^a^	0.16 ± 0.00 ^b^	0.14 ± 0.03 ^b^	0.00
C22:5 n3	1.71 ± 0.10 ^a^	0.94 ± 0.03 ^b^	1.07 ± 0.12 ^b^	0.00
C22:6 n3	17.38 ± 2.65 ^a^	1.39 ± 0.00 ^b^	1.74 ± 0.22 ^b^	0.00
∑SFA	31.20 ± 0.56 ^b^	38.67 ± 0.09 ^a^	34.77 ± 0.09 ^a^	0.00
∑MUFA	30.57 ± 1.65 ^b^	36.27 ± 0.09 ^a^	39.83 ± 0.84 ^a^	0.00
∑PUFA	38.23 ± 2.18 ^a^	25.07 ± 0.12 ^b^	25.33 ± 0.91 ^b^	0.00
∑n-3PUFA	24.10 ± 2.81 ^a^	7.27 ± 0.07 ^b^	7.37 ± 0.37 ^b^	0.00
∑n-6PUFA	13.88 ± 0.19 ^a^	17.56 ± 0.98 ^a^	17.76 ± 0.29 ^a^	0.06
∑n-3/n-6PUFA	5.58 ± 0.43 ^a^	2.59 ± 2.58 ^b^	2.21 ± 2.20 ^b^	0.00

**Table 4:** Sensory attributes of the fillets of barramundi fed with a mixture of poultry by-product meal (PBM), full-fat *Hermetia illucens* (FHI), and partially defatted *Hermetia illucens* (DHI) larvae meal for the complete replacement of fishmeal (FM). The values are the means of nine biological replicates ± the standard error (SE). Means with different superscript letters within the same row indicate significant differences, while means with no superscript letters indicate no variation among the treatments. Means were compared using a one-way ANOVA with Tukey’s post-hoc multiple comparisons test at *p* < 0.05.

Sensory Parameters	0PBM-0HI	70PBM-30FHI	70PBM-30DHI	*p*-Value
Raw visual appearance	3.64 ± 0.41 ^b^	5.88 ± 0.46 ^a^	6.20 ± 0.58 ^a^	0.00
Raw odour	3.56 ± 0.29 ^b^	5.90 ± 0.63 ^a^	5.89 ± 0.55 ^a^	0.00
Raw overall quality	3.67 ± 0.37 ^b^	5.57 ± 0.55 ^ab^	6.10 ± 0.67 ^a^	0.01
Cooked visual appearance	3.93 ± 0.58	4.80 ± 0.76	4.66 ± 0.70	0.65
Cooked odour	3.55 ± 0.36 ^b^	6.21 ± 0.68 ^a^	5.71 ± 0.39 ^a^	0.00
Cooked texture	4.90 ± 0.61	6.05 ± 0.60	6.33 ± 0.63	0.24
Cooked taste	3.99 ± 0.76	5.42 ± 0.80	6.33 ± 0.88	0.15
Cooked overall quality	3.48 ± 0.72	5.43 ± 0.79	6.02 ± 0.82	0.07

**Table 5:** Results for the sensory parameters evaluated using the quality index (QI) method, showing the skin brightness, appearance transparency, flesh texture, flesh blood, flesh odor, and flesh gaping values for barramundi fillets (*n* = 6) from fish fed PBM-HI-based diets during chilled storage. Diet, D; storage time, T. Different superscripts capital letters indicate significant difference between the storage days.

		Test Diets			Two-Way ANOVA		
		0PBM-0HI	70PBM-30FHI	70PBM-30DHI	D	T	D × T
Skin brightness	Day 1	0.00 ± 0.00 ^B^	0.00 ± 0.00 ^B^	0.00 ± 0.00 ^B^			
	Day 4	0.00 ± 0.00 ^B^	0.00 ± 0.00 ^B^	0.00 ± 0.00 ^B^	0.33	0.00	0.34
	Day 8	1.33 ± 0.21 ^A^	1.00 ± 0.00 ^A^	1.17 ± 0.17 ^A^			
Appearance transparency	Day 1	0.00 ± 0.00 ^C^	0.00 ± 0.00 ^C^	0.00 ± 0.00 ^C^			
	Day 4	0.50 ± 0.22 ^B^	0.33 ± 0.21 ^B^	0.50 ± 0.22 ^B^	0.83	0.00	0.94
	Day 8	1.00 ± 0.00 ^A^	1.00 ± 0.00 ^A^	1.00 ± 0.00 ^A^			
Flesh texture	Day 1	0.17 ± 0.17 ^B^	0.00 ± 0.00 ^B^	0.17 ± 0.17 ^B^			
	Day 4	0.83 ± 0.17 ^A^	0.83 ± 0.17 ^A^	0.67 ± 0.21 ^A^	0.83	0.00	0.77
	Day 8	1.00 ± 0.00 ^A^	1.00 ± 0.00 ^A^	1.00 ± 0.00 ^A^			
Flesh blood	Day 1	0.17 ± 0.16 ^C^	0.00 ± 0.00 ^C^	0.00 ± 0.00 ^C^			
	Day 4	1.00 ± 0.00 ^B^	1.50 ± 0.10 ^B^	1.17 ± 0.13 ^B^	0.22	0.00	0.20
	Day 8	1.83 ± 0.15 ^A^	2.00 ± 0.21 ^A^	1.83 ± 0.14 ^A^			
Flesh odour	Day 1	0.00 ± 0.00 ^C^	0.00 ± 0.00 ^C^	0.00 ± 0.00 ^C^			
	Day 4	1.00 ± 0.00 ^B^	1.00 ± 0.22 ^B^	1.00 ± 0.17 ^B^	0.38	0.00	0.42
	Day 8	1.83 ± 0.17 ^A^	2.00 ± 0.00 ^A^	2.00 ± 0.00 ^A^			
Flesh gaping	Day 1	1.50 ± 0.22 ^B^	1.67 ± 0.21 ^B^	1.17 ± 0.40 ^B^			
	Day 4	2.00 ± 0.00 ^A^	1.67 ± 0.21 ^A^	1.83 ± 0.41 ^A^	0.56	0.00	0.43
	Day 8	2.00 ± 0.00 ^A^	2.00 ± 0.00 ^A^	2.00 ± 0.00 ^A^			

## Data Availability

The original contributions presented in the study are included in the article; further inquiries can be directed to the corresponding author.
